# Supplementary material for: An miRNA fingerprint using neural-enriched extracellular vesicles from blood plasma: towards a biomarker for amyotrophic lateral sclerosis/motor neuron disease
Source: Open Biol. 2020 Jun 24;10(6):200116. doi: 10.1098/rsob.200116 (PMC7333885; doi:10.1098/rsob.200116)
Supplement: Banack SA, Dunlop RA, Cox PA, 2020, Identification of a miRNA fingerprint using neural-enriched extracellular vesicles from blood plasma: toward a biomarker for amyotrophic lateral sclerosis/ motor neuron disease (ALS/MND), Royal Society Open Biology, DOI 10.1098/rsob.20200116 [file rsob200116supp1.pdf]

Banack SA, Dunlop RA, Cox PA, 2020, Identification of a miRNA fingerprint using neural-enriched extracellular vesicles from blood plasma: toward a biomarker for amyotrophic lateral sclerosis/ motor neuron disease (ALS/MND), Royal Society Open Biology, DOI 10.1098/rsob.20200116

Target sequences of eight identified miRNA useful to differentiate ALS/MND patients from controls.

**miRNA number**	**miRNA ID**	**Target sequence**
1	hsa-miR-146a-5p	UGAGAACUGAAUUCCAUGGGUU
2	hsa-miR-199a-3p	ACAGUAGUCUGCACAUUGGUUA
3	hsa-miR-4454	GGAUCCGAGUCACGGCACCA
4	hsa-miR-10b-5p	UACCCUGUAGAACCGAAUUUGUG
5	hsa-miR-29b-3p	UAGCACCAUUUGAAAUCAGUGUU
6	hsa-miR-151a-3p	CUAGACUGAAGCUCCUUGAGG
7	hsa-miR-151a-5p	UCGAGGAGCUCACAGUCUAGU
8	hsa-miR-199a-5p	CCCAGUGUUCAGACUACCUGUUC
